# Impact of Albumin and Amino Acids Replacement Therapy, and Protein-Rich Nutrition on Pressure Ulcer Healing in Malnourished Geriatric and Palliative Patients: A Multidisciplinary Clinical-Laboratory Study

**DOI:** 10.3390/jcm15124764

**Published:** 2026-06-18

**Authors:** Lenche Neloska, Katerina Damevska, Ordanche Ribarski, Predrag Kovacevic

**Affiliations:** 1 PHI Specialized Hospital for Geriatric and Palliative Medicine “13 November”, 1000 Skopje, North Macedonia; ordanche_ribarski@yahoo.com; 2 University Clinic for Dermatology, 1000 Skopje, North Macedonia; kate_damevska@yahoo.com; 3 Clinic for Plastic and Reconstructive Surgery, University Clinical Centre Niš, 18000 Niš, Serbia; tpkovacevic@eunet.rs

**Keywords:** pressure ulcers, replacement therapy, malnutrition, total proteins, PUSH score, albumin replacement, geriatric care, wound healing

## Abstract

**Background:** In elderly patients with hypoalbuminaemia, hypoproteinaemia and advanced-stage PUs, chronic inflammation and wound-related protein loss contribute to a self-perpetuating *circulus vitiosus*, in which protein depletion drives deterioration of tissue repair processes, and in turn, ongoing wound-related catabolism further amplifies systemic protein loss. In this context, reduced serum albumin and total protein represent integrated indicators of systemic inflammatory and catabolic burden associated with delayed wound healing. **Aim:** This study evaluated the association between individualized nutritional replacement therapy and pressure ulcer healing in malnourished geriatric and palliative patients, using serum albumin, total protein, and PUSH score as longitudinal outcome indicators. **Methods:** A total of 78 malnourished geriatric and palliative patients with PUs, multiple comorbidities, and poor nutritional status (hypoalbuminemia and/or hypoproteinaemia) receiving patient-tailored nutritional replacement therapy participated in this study. PU assessment using the PUSH version 3.0 tool, as well as measurements of serum albumin and total protein concentrations, were performed on days 0, 30, 60, and 90. **Results:** Our study demonstrates significant improvement in the serum albumin levels, from 30.2 ± 6.19 at baseline to 42.1 ± 5.59 at day 90. Similarly, total protein concentrations increased from 57.8 ± 9.66 at baseline to 70.6 ± 7.03 at day 90. The improvement in protein status was accompanied by a significant reduction in the PUSH score, from 10.9 ± 2.94 at the first assessment to 2.9 ± 2.63 at the final assessment. Spearman’s rank-order correlation analysis between serum albumin, total protein, and PUSH score demonstrated a significant moderate inverse correlation at later assessment points (day 60 and 90). **Conclusions:** Individualized and targeted replacement therapy was associated with improved protein status and reduced pressure ulcer severity. Increases in serum albumin and total protein paralleled a marked reduction in PUSH scores, suggesting attenuation of the inflammatory-catabolic *circulus vitiosus* and a progressive shift toward wound healing in geriatric and palliative patients.

## 1. Introduction

Pressure injuries/ulcers (PUs) are localized damage to the skin and/or underlying tissue, usually over a bony prominence or related to medical or other devices, resulting from prolonged pressure [1]. As a critical healthcare challenge, PUs have a high prevalence up to 29% among malnourished geriatric patients with comorbidities in long-term facilities [1,2].

Age-related physiological decline, comorbidities, immobility, chronic inflammation (i.e., inflammaging), tissue vulnerability, and malnutrition, as well as pressure, shear, and friction, significantly increase the susceptibility to tissue breakdown and development of PU [3,4].

Population aging has a significant impact on the healthcare system in North Macedonia, where the proportion of adults aged ≥ 65 years increased from 13.1% in 2013 to 18.1% in 2023, according to the data from the State Statistical Office [5].

Considering the substantial burden of PUs on healthcare systems, particularly in the context of the rapidly growing elderly population, as well as their devastating effects on overall health and quality of life [4], patient-tailored nutritional intervention should be considered a top priority in the management of malnourished patients with hypoalbuminemia and hypoproteinaemia and PUs.

Total protein and serum albumin are laboratory indicators reflecting the interplay between nutritional status, systemic inflammation, and catabolic burden during pressure ulcer (PU) healing. In elderly malnourished and severely malnourished patients with advanced-stage PUs, chronic inflammation and wound-related protein loss contribute to a self-perpetuating *circulus vitiosus*, in which protein depletion leads to dysfunction of tissue repair processes, and in turn, ongoing wound-related catabolism further amplifies systemic protein loss. This persistent metabolic imbalance is associated with impaired fibroblast activity, reduced angiogenesis during the proliferative phase, and decreased collagen synthesis during tissue remodeling, ultimately contributing to delayed wound healing. In this regard, serum albumin and total protein represent integrated indicators of systemic inflammatory and catabolic burden associated with poor healing trajectories rather than isolated nutritional deficits [2,6,7,8,9,10].

This study aims to evaluate the association between protein status, as reflected by serum albumin and total protein levels, and pressure ulcer healing outcomes in malnourished and severely malnourished geriatric and palliative patients, within the context of a self-perpetuating *circulus vitiosus* linking inflammation, catabolism, and impaired tissue repair, and to assess the impact of patient-tailored nutritional replacement therapy and protein-rich nutrition on this process.

## 2. Materials and Methods

### 2.1. Patient Selection

**Figure 1:**
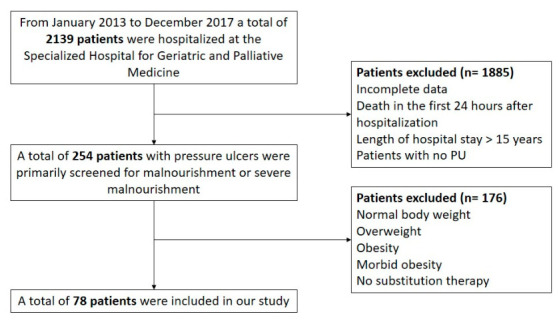
Patient recruitment with inclusion and exclusion criteria.

This prospective cohort study included 78 malnourished or severely malnourished geriatric and palliative patients with pressure ulcers admitted to the Specialized Hospital for Geriatric and Palliative Medicine in Skopje. The eligibility criteria, including the inclusion and exclusion criteria for patient recruitment, are presented in Figure 1. Nutritional screening of participants was performed using the Mini Nutritional Assessment-Short Form (MNA-SF). The study was conducted in accordance with the Declaration of Helsinki and approved by the Institutional Review Board of Specialized Hospital for Geriatric and Palliative Medicine in Skopje, Republic of North Macedonia.

### 2.2. Laboratory Analysis of Serum Albumin and Total Proteins

Biomarkers assessment

For the purposes of this research, approximately 5 mL of venous blood was collected from each participant in a vacuum tube with clot activator on days 0, 30, 60, and 90. After clotting and centrifugation, the serum was used for the spectrophotometric determination of serum albumin and total protein concentration. Within the framework of this study, the reference range for serum albumin was defined as 35–50 g/L, while for total proteins it was defined as 65–80 g/L.

### 2.3. Pressure Ulcer Assessment

Pressure ulcer healing was assessed on days 0, 30, 60, and 90 using the PUSH version 3.0 tool in accordance with the recommendations of the European Pressure Ulcer Advisory Panel (EPUAP). The PUSH tool evaluates the ulcer surface area, exudate amount, and type of tissue present in the ulcer bed. The cumulative PUSH score, determined from assessment of the aforementioned wound characteristics, ranges from 0, indicating complete wound healing, to 17, representing a severe pressure ulcer with poor healing status [11].

### 2.4. Patient-Tailored Nutritional Replacement Therapy Administration of Human Albumin, Amino Acid Replacement Therapy, and Protein-Rich Nutrition

To address hypoalbuminaemia and/or hypoproteinaemia in our cohort, individualized targeted human albumin and/or amino acid replacement therapy or protein-rich nutrition was implemented based on each patient’s comorbidities, polypharmacy, nutritional status, and pressure ulcer’s stage. Particular consideration was given to increased protein loss through wound exudate and inflammation-mediated reduced hepatic albumin synthesis. In the context of protein-rich nutrition and targeted protein–amino acid replacement therapy in our study, we used either 20% Human Albumin infusion CSL BEHRING GmbH, Marburg, Germany (50 mL), amino acid infusion (10% Aminoven^®^ 1000 mL Fresenius Kabi Austria GmbH, Graz, Austria), protein-rich supplement (Nutricia FortiCare^®^ 125 mL, Zoetermeer, The Netherlands), or a combination of them.

### 2.5. Statistical Analysis

The obtained data were analyzed using descriptive statistical methods. Categorical variables, such as gender/sex, age, source of admission, comorbidities, chronic therapy, types of patient-tailored nutritional replacement therapy as well as PU anatomical localization and number of PU per patient, were summarized as absolute counts and percentages. Differences in serum albumin, total protein levels, and PUSH score measured at the four assessments (day 0, 30, 60, and 90) were evaluated using the Friedman analysis of variance by ranks (Friedman ANOVA Chi Sqr. Test). A *p*-value < 0.05 was considered statistically significant. Additionally, Spearman’s rank-order correlation analysis was used to evaluate the correlation between serum albumin, total protein levels, and PUSH score.

## 3. Results

### 3.1. Patient’s Demographic and Clinical Characteristics

A total of 78 malnourished or severely malnourished geriatric and palliative patients, with hypoalbuminaemia—hypoproteinaemia—participated in our study, with a predominance of female patients. The mean age in our study sample is 76.1 ± 12.09 years (range 38–95 years). The majority of patients came from their homes, while fewer came from the internal medicine or surgical departments or other healthcare institutions. Cardiomyopathy and hypertension are the most prevalent of the chronic diseases. In terms of chronic therapy, cardiovascular and antimicrobial therapies were the most common, which is consistent with the high prevalence of chronic cardiovascular disease and pressure ulcers in this cohort. Table 1 provides a more detailed overview of the participants’ demographic and clinical characteristics relevant to this research.

In terms of PU, the total number of PU in our study population was 112. The most frequent anatomical sites of PU were regio sacralis, regio trochanterica, and regio calcanea. In contrast, other anatomical sites, such as regio glutea, regio scapularis, regio vertebralis, regio malleolaris, and regio pedis, were less frequently registered. The majority of patients had a single PU, whereas fewer patients had multiple PUs (Table 2).

### 3.2. Patient-Tailored Nutritional Replacement Therapy

Patient-tailored nutritional replacement therapy was administered according to a multidisciplinary clinical assessment of the patient’s protein status, including serum albumin and total protein levels, comorbidities, other laboratory parameters, and PU characteristics. The majority of patients received combined nutritional replacement therapy, with 65.38% of patients treated with a combination of 20% human albumin infusion and 10% amino acid infusion. Single nutritional replacement therapy was less frequent, with protein-rich nutrition in 14.10% of patients, 20% human albumin infusion in 10.26% of patients, and 10% amino acid infusion alone in 1.28%. Additionally, 8.98% of patients received a combination of 10% amino acid infusion and protein-rich nutrition (Table 3).

### 3.3. Serum Albumin and Total Protein Levels

Although comprehensive laboratory panels were assessed for each patient, our study primarily focused on total protein and serum albumin over the 90-day follow-up period (Table 4).

Total protein levels demonstrated a progressive improvement throughout the 90-day follow-up period, indicating a gradual improvement in the protein status. The highest cumulative values of total protein were observed at the end of the study (70.6 ± 7.03). Statistical analysis confirmed a significant difference in the total protein levels across the time assessment points, suggesting a positive trend over time (Friedman ANOVA Chi Sqr. (Nn = 78, df = 3) = 234.0000, *p* = 0.00000).

Similarly, serum albumin levels demonstrated a progressive increase during the 90-day follow-up, with the highest values recorded at day 90 (42.1 ± 5.59). The observed changes at the assessment time-points were statistically significant, indicating a favorable response to the personalized nutritional replacement therapy (Friedman ANOVA Chi Sqr. (N n = 78, df = 3) = 232.8154, *p* = 0.00000).

### 3.4. PUSH Score

Over the 90-day follow-up period, our data showed a progressive decrease in the mean PUSH score, indicating a continuous improvement in the PU healing (Table 4). The greatest reduction was observed at the final assessment (2.9 ± 2.63), reflecting substantial PU healing over time. Statistical analysis demonstrated a significant difference in the PUSH score across all assessment points (Friedman ANOVA Chi Sqr. (Nn = 78, df = 3) = 222.5868, *p* = 0.00000).

### 3.5. Correlation Between PUSH Score, Serum Albumin and Total Protein Levels

Correlation analysis revealed an inverse relationship between the PU severity, as measured by the PUSH score, and the patient’s serum albumin and total protein levels. Higher total protein and serum albumin levels were usually associated with a lower PUSH score, indicating a favorable PU healing outcome (Table 5).

The association between total protein levels and PUSH score was weak during most of the follow-up period, becoming statistically significant at the final assessment point (day 90). On the other hand, the relationship between serum albumin and PUSH score strengthened over time, with a significant moderate inverse correlation observed during the later stages of the follow-up (day 60 and 90). These findings suggest that improvements in the serum albumin and total protein levels were associated with enhanced PU healing, supporting their role as important factors in the healing process of PUs.

### 3.6. Dynamics of PU Healing During the 90-Day Follow-Up Period

During the 90-day follow-up period, a progressive shift from most-severe towards less-severe PU stages was observed, indicating substantial PU healing and overall clinical improvement among the total of 112 evaluated PUs. There was no progression in terms of migration from lower to higher stages. At baseline (day 0), the majority of PUs were classified as advanced-stage PU (89.29%), of which with stage III predominating (58.04%), followed by stage IV (21.43), stage V (8.04) stage VI (1.78), indicating severe skin damage (Table 6).

At day 30, reduced severity and PU stage migration from most-severe to less-severe stages were observed. Hence, a progressive reduction in advanced stages (stage IV, V, and VI) from 31.25% to 19.64% was observed. A decrease in the proportion of stage IV (17.86%) and V (1.78%), accompanied by an increase in proportion in stage III (58.93%), and stage II (21.43%) was observed. By day 60, stage I PUs were recorded, following the trend of continuous PU reduction from most severe to less severe stages, suggesting successful wound healing and regression in the PU severity. Moreover, by day 60, an increase in the number of stage II PUs (33.93%) was observed, accompanied by a reduction in stage III (42.86%) and stage IV PUs (1.78%) compared with day 30. Furthermore, at day 90, complete skin resuscitation and healing were documented in a substantial proportion of the evaluated PUs (21.43%), indicating an absence of PU (stage 0). At the same time, the majority of evaluated PUs were classified as either stage I or II, whereas a smaller proportion of PUs (16.07%) were classified as stage III.

## 4. Discussion

In this single-center study of malnourished and severely malnourished geriatric and palliative patients with hypoalbuminaemia and hypoproteinaemia and PUs, significant improvement in PU healing was observed over the 90-day observation. These clinical outcomes, reflected by a decrease in the PUSH score, were paralleled by an increase in serum albumin and total protein concentrations, indicating an overall improvement in protein status and clinical recovery. Moreover, the observed inverse relationship between the PUSH score on the one hand, and the serum albumin and total protein on the other, suggests that improvement in the protein status may contribute to more successful PU healing.

Latest guidelines for PU management emphasize the importance of adequate nutritional support in geriatric patients, with minimum requirements of 30–35 kcal/kg/day and 1.25–1.5 g/kg/day protein intake [8]. To meet the nutritional requirements of malnourished geriatric patients, up to 2.0 g/kg/day protein intake can be considered [12,13].

Serum albumin and Total proteins are widely available, cost-effective biomarkers that are commonly used in nutritional assessment, although their interpretation requires few precautions in surgery patients, trauma patients, or anorexia nervosa [14].

Hypoalbuminaemia and hypoproteinaemia, are associated with pressure ulcer development and impaired healing and may reflect the combined effects of malnutrition, systemic inflammation, and increased catabolic activity. Low baseline levels of total protein and serum albumin serve as indicators of malnutrition, muscle wasting, and localized tissue frailty, effectively predicting the imminent development of PUs [8,15,16]. Protein depletion, reflected by hypoalbuminaemia and hypoproteinaemia, is associated with reduced fibroblast cell activity, impaired angiogenesis during the proliferative phase of PU healing, and reduced collagen synthesis during the tissue remodeling. Furthermore, hypoproteinaemia is exacerbated by protein loss through wound exudation, which can reach up to 100 g per day [2].

Pressure ulcer-induced chronic systemic inflammation leads to the upregulation of inflammatory cytokines (IL-1β, IL-6, and TNF-α). Serum albumin levels are directly modulated by these inflammatory cytokines. These cytokines exert a dual effect on hepatic protein synthesis: they promote the upregulation of C-reactive protein (CRP), while concurrently impairing albumin production and accelerating albumin degradation, hence reducing circulating serum albumin concentrations [9,17]. Inflammatory cytokines can also negatively affect food intake by suppressing the appetite-regulating center in the central nervous system (CNS) [13].

Furthermore, hypoalbuminaemia in patients with pressure ulcers is multifactorial and may reflect not only inflammation, but also malnutrition, increased metabolic demands, protein loss through wound exudate, altered albumin distribution, and increased albumin catabolism. Consequently, serum albumin should be interpreted as a composite biomarker of nutritional and inflammatory status, rather than a purely nutritional marker. In addition, while lower serum albumin and total protein in malnourished patients are associated with increased susceptibility of PU development and impaired healing, the PU itself may further reduce circulating protein levels through wound exudation and inflammation-mediated suppression of protein synthesis [7,18,19].

In malnourished and severely malnourished elderly patients with advanced-stage PUs, chronic inflammation and wound exudate-related protein loss contribute to a self-perpetuating *circulus vitiosus*, in which protein depletion leads to dysfunction of tissue repair processes, and in turn, ongoing wound-related catabolism further amplifies systemic protein loss. Hence, an exudative wound acts as a site of continuous, massive protein loss [20]. Therefore, serum albumin and total protein were included as clinically relevant biomarkers reflecting nutritional status, inflammatory burden, and overall physiological reserve.

Gupta et al. emphasize the substantial impact of hypoalbuminaemia, along with advanced age, anemia, and chronic inflammation, on pressure ulcer outcomes [21]. In addition to proteins, studies emphasize that additional nutritional factors, such as vitamins, antioxidants, zinc, and lipids, may also play an important role in the PU healing [1,3,22]. Cereda et al. demonstrate that arginine, vitamin C, and zinc also have beneficial effects on pressure ulcer healing [1], while Desneves et al. demonstrated a significant decline in the PUSH score with adequate amino acid and micronutrient optimization [23]. Liu P et al. demonstrated that arginine-enriched enteral formulas were associated with improved PU healing outcomes in both malnourished and non-malnourished patients [24]. As a semi-essential amino acid, arginine may enhance immunity and collagen synthesis, therefore promoting wound regeneration [24]. Similarly, the systematic review by Moran JM et al., which included 14 clinical trials, reported that nutritional optimization did not reduce the incidence but significantly limited PU progression and severity in patients with hip fractures [25]. The study by van Anholt et al. focused primarily on nutritional support in non-malnourished patients with PU and demonstrated that oral supplementation enriched with protein, arginine, and micronutrients correlates with improved PU healing and reduced wound severity [26]. These findings are consistent with our results that improvements in patients’ protein status correlate with reduced wound severity and improved wound-healing outcomes.

Serum albumin is utilized as a biomarker, reflecting both nutritional and inflammatory status, in other clinical studies in relation to other chronic diseases. Although, Ichim et al. did not find significant changes in serum albumin levels following fecal microbiota transplantation in patients with alcohol-associated liver cirrhosis, serum albumin remained one of the principal measures used to monitor treatment response and disease progression [27].

For the purposes of our study, the Pressure Ulcer Scale for Healing (PUSH score) was used as the primary instrument to assess pressure ulcer healing over 90 days. The National Pressure Ulcer Advisory Panel (NPUAP) introduced the PUSH score as a cost-effective, practical instrument for daily clinical practice in chronic wound assessment in 1997 [28]. The PUSH score has been widely adopted in clinical use since 2001. The findings from our study support the practical use of the PUSH score in PU assessment, with a mean PUSH score reduction from 10.9 ± 2.94 at baseline to 2.9 ± 2.63 at final assessment.

### Limitations and Future Perspectives

This study has several limitations that should be considered when interpreting the results. Firstly, it is a single-center study, which may limit the applicability of our findings to other healthcare settings. Secondly, this study included a relatively small cohort of malnourished geriatric and palliative patients with multiple comorbidities, polypharmacy and pressure ulcers, which may have limited the statistical power of the analysis.

Future metacentric cohort studies with larger sample sizes are essential to further investigate the relationship between the serum albumin, total protein concentrations and the PUSH score.

## 5. Conclusions

The present findings demonstrate that individualized and targeted nutritional replacement therapy and/or protein-rich nutrition play an important role in pressure ulcer healing outcomes. Increases in serum albumin and total protein paralleled a marked reduction in PUSH scores; reduced wound stage, severity and progression; and improved overall clinical outcomes, suggesting attenuation of the inflammatory-catabolic *circulus vitiosus* and a progressive shift toward wound healing in geriatric and palliative patients.

## Figures and Tables

**Table 1:** Patient’s demographic and clinical characteristics.

Gender	n (%)	Difference Test
Male	35 (44.9)	*p* = 0.2027
Female	43 (55.1)	
**Mean age, SD, range**	76.1 ± 12.09 (38–95)	
**The patient comes from**		
Own home	41 (52.6)	*p* = 0.000
Surgical hospital	16 (20.5)	
Internal Medicine Department	18 (23.1)	
Other	3 (3.8)	
**Chronic therapy**		
Cardiovascular therapy	67 (85.9)	
Anti-anemic therapy	39 (50.0)	
Antimicrobial therapy	73 (93.6)	
Anti-edema therapy	3 (3.8)	
Diuretic therapy	28 (35.9)	
Anti-diabetic therapy	16 (20.5)	
Vasoprotective therapy	22 (28.2)	
Analgesic therapy	48 (61.5)	
Gastroprotective therapy	36 (46.2)	
Anti-hemorrhagic therapy	10 (12.8)	
Neurological therapy	28 (35.9)	
Psychiatric therapy	31 (39.7)	
Anticoagulant/antiplatelet therapy	18 (23.1)	
Corticosteroid therapy	40 (51.3)	
Antineoplastic therapy	6 (7.7)	
Lipid-lowering therapy	5 (6.4)	
Antihistamine therapy	14 (17.9)	
Laxative and probiotic therapy	12 (15.4)	
Antispasmodic therapy	6 (7.7)	
Bronchodilator therapy	19 (24.4)	
**Mean value ± SD, range**	**6.7 ± 12.09 (2–12)**	
**Chronic diseases**		
Diabetes	13 (16.7)	
Cerebrovascular diseases	17 (21.6)	
Cardiomyopathy	52 (66.7)	
Hypertension	41 (52.6)	
Anemia	4 (5.1)	
COPD	5 (6.4)	
Neurological diseases	20 (25.6)	
Psychiatric diseases	12 (15.4)	
Kidney diseases	3 (3.8)	
Liver diseases	8 (10.3)	
Oncological diseases	7 (9.0)	
**Mean value ± SD, range**	**2.3 ± 1.19 (0–6)**	

**Table 2:** Distribution of PU according to anatomical localization and number of PU per patient at baseline (day 0).

Number of PU per Patient	n (%)
1	51 (45.5)
2	20 (17.9)
3	7 (6.3)
Total number of PU	112
**Anatomical Localization of PU, n (%)**	
Regio sacralis	47 (42.0)
Regio glutea	12 (10.7)
Regio trochanterica	19 (17.0)
Regio calcanea	16 (14.3)
Regio scapularis	3 (2.7)
Regio vertebralis	3 (2.7)
Regio malleolaris	4 (3.6)
Regio pedis	8 (7.14)

**Table 3:** Patient-tailored nutritional replacement therapy.

Singe Replacement Therapy	n (%)
20% Human albumin infusion	8 (10.26)
10% Amino acid infusion	1 (1.28)
Protein-rich nutrition	11 (14.10)
**Combined replacement therapy**	n (%)
20% Human albumin infusion + 10% Amino acid infusion	51 (65.38)
10% Amino acid infusion + Protein-rich nutrition	7 (8.98)

**Table 4:** Mean values of serum albumin, total protein, and PUSH score at day 0, 30, 60, and 90.

Day	PUSH Score Mean ± SD	Total Protein Mean ± SD	Serum Albumin Mean ± SD
0	10.9 ± 2.94	57.8 ± 9.66	30.2 ± 6.19
30	8.9 ± 2.86	61.3 ± 9.66	33.7 ± 6.19
60	5.9 ± 2.86	65.2 ± 7.08	37.3 ± 5.49
90	2.9 ± 2.63	70.6 ± 7.03	42.1 ± 5.59

**Table 5:** Spearman Rank Order Correlations between serum albumin, total protein and PUSH score.

Day 0	Total Protein	Serum Albumin
PUSH score	−0.018510	−0.187716
**Day 30**	**Total protein**	**Serum albumin**
PUSH score	−0.108540	−0.217353
**Day 60**	**Total protein**	**Serum albumin**
PUSH score	−0.158856	−0.383896
**Day 90**	**Total protein**	**Serum albumin**
PUSH score	−0.293325	−0.439972

**Table 6:** Dynamics of PUs healing over the 90-day follow-up period.

PU Stage	Day 0 n (%)	Day 30 n (%)	Day 60 n (%)	Day 90 n (%)
0				24 (21.43)
I			24 (21.43)	40 (35.71)
II	12 (10.71)	24 (21.43)	38 (33.93)	30 (26.79)
III	65 (58.04)	66 (58.93)	48 (42.86)	18 (16.07)
IV	24 (21.43)	20 (17.86)	2 (1.78)	
V	9 (8.04)	2 (1.78)		
VI	2 (1.78)			

## Data Availability

The data analyzed in this study were provided by the PHI Specialized Hospital for Geriatric and Palliative Medicine, “13 November”-Skopje, Republic of North Macedonia. Due to the institution’s policies, the datasets are not publicly available.

## Abbreviations

The following abbreviations are used in this manuscript:

PU	Pressure ulcer
PUSH	Pressure Ulcer Scale for Healing
MNA-SF	Mini Nutritional Assessment-Short Form
CRP	C-Reactive Protein
TNFα	Tumor Necrosis Factor-alpha
IL-6	Interleukin-6
IL-1β	Interleukin-1 beta
CNS	Central Nervous system
